# The effect of aerobic exercise on cerebral perfusion in patients with vascular cognitive impairment, the Excersion-VCI randomised controlled clinical trial

**DOI:** 10.1016/j.cccb.2025.100386

**Published:** 2025-05-24

**Authors:** Liz R. van Hout, Justine Moonen, Annebet E. Leeuwis, Juliette van Alphen, Mathijs Dijsselhof, Raquel P. Amier, Frederik Barkhof, Esther E. Bron, Doeschka A. Ferro, Alexander G.J. Harms, Rosalie J. Huijsmans, Joost P.A. Kuijer, Sanne Kuipers, Matthias J.P. van Osch, Niels D. Prins, Marc B. Rietberg, Charlotte E. Teunissen, Henk Jan Mutsaerts, Geert Jan Biessels, Wiesje M. van der Flier, on behalf of the Heart-Brain Connection Consortium

**Affiliations:** a Alzheimer Centre Amsterdam, Neurology, Vrije Universiteit Amsterdam, Amsterdam UMC location VUmc, Amsterdam, The Netherlands; b Amsterdam Neuroscience, Neurodegeneration, Amsterdam, The Netherlands; c Department of Human Movement Sciences, Faculty of Behavioural and Movement Sciences, Vrije Universiteit Amsterdam, The Netherlands; d Department of Radiology and Nuclear Medicine, Amsterdam UMC, VU University Medical Centre, Amsterdam, The Netherlands; e Department of Cardiology, VU University Medical Centre, Amsterdam, The Netherlands; f Institute of Healthcare Engineering, University College London, London, UK; Institute of Neurology, University College London, London, UK; g Biomedical Imaging Group Rotterdam, Department of Radiology & Nuclear Medicine, Erasmus MC University Medical Centre Rotterdam, The Netherlands; h Department of Neurology, University Medical Centre Utrecht, Utrecht, The Netherlands; i Department of Rehabilitation, Amsterdam UMC, location VUmc, Amsterdam, The Netherlands; j C.J. Gorter MRI Centre, Department of Radiology, Leiden University Medical Centre, Leiden, The Netherlands; k Brain Research Centre, Amsterdam, The Netherlands; l Neurochemistry Laboratory, Department of Clinical Chemistry

**Keywords:** Vascular cognitive impairment, Cerebral blood flow, Exercise, Randomised clinical trial, Arterial spin labelling, cognition

## Abstract

Aerobic exercise may improve cerebral perfusion and may thereby attenuate, or delay, cognitive decline. Excersion-VCI aimed to evaluate the effect of aerobic exercise on cerebral perfusion in patients with vascular cognitive impairment (VCI).

This Randomized Controlled Trial included non-demented adults ≥50 years diagnosed with VCI. Patients were randomly assigned either home-based aerobic interval training (exercise group) or information sessions (control group). Primary outcome was change in Arterial Spin Labelling MRI grey matter cerebral perfusion from baseline to 14-week follow-up. Per-protocol analysis was performed in patients who completed the follow-up. Secondary outcomes were VO_2max_ and cognitive function. Exploratory outcomes were depression and apathy, White Matter Hyperintensities, cerebral volumes, and blood biomarkers.

Fifty-eight VCI patients (mean age 67.0 ± 6.7 years) were allocated to the exercise (*n* = 28) or control group (*n* = 30). Intention-to-treat analyses showed that change in grey matter cerebral perfusion in the exercise group did not differ from the control group (*p* = 0.38), nor were there group differences in change in VO2max (*p* = 0.17). The exercise group showed an increase in triglycerides compared to the control group (*p* = 0.04). No group differences were found for other outcomes. Per protocol analyses showed improvement in VO_2max_ in the exercise group compared to the control group (*p* = 0.04).

An aerobic exercise program in VCI patients improved cardiorespiratory fitness in those who adhered to the protocol, but did not show significant effects on grey matter cerebral perfusion or other outcomes. The intervention duration of 14-weeks may have been too short to measure changes in perfusion or improvements in cognitive function.

## Highlights

• This RCT in vascular cognitive impairment investigated the effect of an exercise intervention.
• VO2max increased in participants who adhered to the exercise intervention protocol.
• The intervention had no effect on total grey matter CBF or cognitive function.

## 1 Introduction

Vascular cognitive impairment (VCI) encompasses all cognitive deficits caused by cerebrovascular damage and explains 20–40 % of dementia diagnoses. Cerebrovascular pathology is also frequently present in other neurodegenerative pathologies such as Alzheimer’s Disease [1]. There is currently no curative treatment, underlining the importance of prevention of VCI [2]. Cerebral hypoperfusion is a driver of the small vessel disease (SVD) pathologies and clinical manifestations of VCI, including presence of microbleeds, periventricular spaces, white matter hyperintensities (WMH), and infarcts [1].

Aerobic exercise may increase cerebral blood flow (CBF) and thereby contribute to prevention of cognitive decline [3]. In an observational study in persons aged >60 years without cognitive impairment a higher level of physical activity has been related to a smaller decline in CBF and cognitive function at 4-years follow-up [4]. Another study in healthy older adults that implemented an aerobic exercise program found positive effects on both cognitive function and CBF at 6 months follow-up [5].

Randomized controlled trials (RCTs) that have investigated the effect of an exercise intervention on CBF in healthy older adults [6], Mild Cognitive Impairment [7], or Alzheimer’s Disease [8] were inconsistent, as they found no effects [8], or a positive effect [6,7]. Those trials that demonstrated a positive effect of aerobic exercise on CBF also showed improvements on varying cognitive domains [6,7]. High-quality evidence of the effect of physical activity in VCI, however, is scarce. One RCT in subcortical ischemic VCI was performed, which found a positive effect of a 6 months exercise program on cognitive function compared to an inactive control group [9]. Next to cognitive function, aerobic exercise may have a positive influence on symptoms of depression and apathy [10], which are common symptoms of VCI.

Aerobic exercise may have a cascade of physiological effects resulting in improved or preserved cognitive function other than effects on cerebral perfusion. Studies have suggested that exercise has a positive impact on metabolic disease [11], inflammation and growth factors [12], and neurodegeneration according to brain volumes [13] or blood biomarkers for Alzheimer’s Disease [3].

This single-blind randomised controlled trial studied the effect of a 14-week aerobic exercise program on grey matter CBF as primary outcome in patients with VCI. We hypothesized that the exercise program would improve cardiorespiratory fitness, and thereby increase CBF. Predefined secondary outcomes were physical fitness and cognitive function. Other exploratory outcome measures were symptoms of depression and apathy, cerebral volumes, and blood based biomarkers.

## 2 Methods

### 2.1 Study design

The Excersion-VCI study was a multi-centre randomised controlled trial with blinded outcome assessment that was part of the Heart-Brain Consortium [14]. Participants were randomised to either an aerobic exercise program or to the control group. Participants underwent screening, baseline, and 14-week follow-up in the clinics. Participants were screened for eligibility and safety for cardiorespiratory fitness measurements. At both baseline and follow-up, MRI, a maximal exercise capacity test, and cognitive and neuropsychiatric function and venipuncture were performed. In addition, cardiac MRI was performed at baseline. In the exercise group the baseline and follow-up were performed within a timeframe of 14 days of the first and last exercise sessions. The medical ethical evaluation board of the Amsterdam UMC and UMC Utrecht approved the study and all patients provided written informed consent.

### 2.2 Participants

A total of 62 patients with VCI were recruited from the outpatient memory clinic of Amsterdam UMC, location VU medical centre (VUmc) (*n* = 43), University Medical Centre Utrecht (UMCU) (*n* = 2), and via the website www.Hersenonderzoek.nl (*n* = 17; of which *n* = 12 were included at Amsterdam UMC and *n* = 5 at UMCU). Inclusion criteria were 1) age ≥50 years, 2) presence of cognitive complaints but no dementia (i.e. Subjective Cognitive Decline or Mild Cognitive Impairment), according to a Mini-Mental State Examination (MMSE) ≥22 and a Clinical Dementia Rating ≤0.5, 3) vascular brain damage on MRI according to the presence of moderate-to-severe WMH (Fazekas scale > 1) and/or (lacunar) infarct(s) and/or intracerebral (micro-) haemorrhage(s); or having only mild WMH (Fazekas 1) and being at increased vascular risk [14]. Exclusion criteria were a diagnosis of dementia, contraindication for MRI, already taking part in an aerobic exercise program or in another trial, or presence of diseases that affect cognition or form a contraindication to undergo an aerobic exercise program (e.g. major neurologic, psychiatric, cardiac, or musculoskeletal diseases).

### 2.3 Randomisation and blinding

After baseline assessment, participants were assigned to either the exercise or the control group using the minimization approach, a method of adaptive stratified sampling. This technique aims to create a balanced distribution between both groups by utilizing prognostic factors [15]. Minimization was executed through the Minim software with a 1:1 allocation ratio and equal weight given to four factors: VCI disease severity (Clinical Dementia Rating 0 vs. 0.5), age (<65 vs. >65 years), gender, and centre [16]. Randomisation was carried out by an independent researcher who was blinded for participants’ identity. The outcome assessor was blind to group allocations.

### 2.4 Intervention

The exercise intervention was an at-home aerobic exercise program on a bicycle ergometer (Kettler Ergometer, E7, Ense, Deutschland) designed to improve cardiorespiratory fitness. The program consisted of 3 exercise sessions with a duration of 48 min per week during 14 weeks, resulting in a total of 42 exercise sessions. Each exercise session was an aerobic interval training (AIT) specified by the 4 × 4-minute interval model [17], as shown in Supplementary figure A.1 [14]. Exercise intensity was individualized by each participant’s peak heart rate (HRpeak). During the exercise sessions participants wore a heart rate monitor providing the bicycle with real time feedback and, subsequently, work rate was automatically adjusted to ensure the intended exercise intensity. Adherence to exercise frequency was measured by self-report. Participants were required to keep a diary on completion of the exercise session (“yes/no”). Thirteen out of 42 sessions were supervised by a buddy (a physical therapist in training) with the goal to keep participants motivated. The control group received two individual information sessions of 45 min providing information about VCI and cardiovascular risk factors.

### 2.5 MRI

#### 2.5.1 MRI acquisition

MRI was performed on a Gemini 3T PET-MR scanner at Amsterdam UMC or a Ingenia 3T scanner at UMC Utrecht (both Philips Healthcare, Best, The Netherlands).

##### 2.5.1.1 Structural MRI

3D T1-weighted turbo field echo (TFE) images were acquired with the following parameters: repetition time (TR)=7.9 ms, Echo Time (TE)=4.5 ms, Flip Angle (FA)=8°, voxel size=1 × 1 × 1 mm^3^. 3D T2-weighted FLAIR images were acquired with TR=4800 ms, TE=279 ms, Inversion time (TI)=1650 ms, FA=90°, voxel size=1 × 1 × 1mm^3.

##### 2.5.1.2 ASL-MRI

A pseudo-continuous ASL (pCASL) MRI sequence was used for CBF quantification, using a background suppressed 2D EPI with a resolution of 4 × 4 × 3.0mm^3, TR=4400 ms, TE=13.9 ms, labelling duration=1800 ms, PLD=1800 ms, PLD range due to 2D slice-by-slice acquisition = 1800–2497 ms, slice readout time=32 ms, accompanied by a 2 s TR M0 image without background suppression and labelling but otherwise identical readout.

#### 2.5.2 Image processing

##### 2.5.2.1 Structural MRI

T1 and T2-FLAIR images were visually assessed by trained staff (MD and FB, respectively 4 and above 30 years of experience) for rating of microbleeds, periventricular spaces, Fazekas scale, white matter hyperintensities, and infarcts according to the STRIVE criteria [18]. The total small vessel disease (SVD) Staals compound score (range 0–4) was computed as a summation of 1) the presence of lacunar infarct(s), 2) presence of lobar or non-lobar microbleed(s), 3) moderate to severe perivascular spaces in the basal ganglia, and 4) a White Matter Hyperintensity Fazekas rating of 3 [19]. The segmented T1-weighted images were used for probabilistic tissue segmentations for grey matter, white matter, and cerebrospinal fluid volumes, using the unified tissue segmentation method [20] from SPM8 (Statistical Para-metric Mapping, London, UK). T1-weighted and FLAIR images were used for white matter hyperintensity (WMH) segmentation, using an automated pipeline (Quantib B.V.) [21]. All tissues were corrected for WMH lesions using the automatic WMH segmentations, and manually segmented lesions that depict infarctions and other pathologies were excluded using patient-specific masks. The manual segmentations were performed by the aforementioned trained staff. Next, from the structural MRI, the secondary outcome measures total grey matter (GM) volume, total white matter (WM) volume, cerebrospinal fluid (CSF) volume, and WMH volume were obtained.

##### 2.5.2.2 ASL-MRI

PCASL single-PLD images were processed using the Iris pipeline as described previously [[22], [23]]. In short, the ROI segmentation was based on the grey matter and white matter segmentations from the T1-weighted scans (SPM8, London, UK) [20], and multi-atlas registration of 30 atlases with 83 structural brain regions [[24], [25]]. In addition, regions were grouped into the four brain lobes (frontal, parietal, occipital, temporal) in each hemisphere (Hammers et al., 2003). As described previously, all subject-specific ROIs exclude manually segmented lesions. ASL quantification into CBF maps was based on a single-compartment model after the subtraction of labelled images from control images [26]. The M0 image was used to scale the signal intensities of the subtracted ASL images to absolute CBF values. Further image processing included motion-correction of the raw ASL data [27] and partial volume correction [28]. CBF values were calculated in the supra-tentorial cerebrum, since the cerebellum was not fully covered.

The outcome measures from the ASL-MRI measurements were total and lobar grey matter CBF and the spatial coefficient of variation (CoV). Spatial CoV was calculated as (SD/mean)*100 over total grey matter, and subsequently log transformed [29]. It can be used as a proxy of arterial transit time, providing information on the cerebral haemodynamics.

#### 2.5.3 Quality control

All MR images were visually checked on incidental findings (i.e. pathologies) and quality by a trained researcher. Images with motion artefacts, incomplete ASL sequences, labelling errors, vascular artefacts (ASL signal predominantly in the major vessels) or unreliably low or high CBF values (i.e. <30ml/100 g/min or >100ml/100 g/min) were excluded from analyses [26,30].

### 2.6 Cardiorespiratory fitness

Cardiorespiratory fitness was assessed with a maximal exercise capacity test (maximum oxygen consumption, i.e. VO2max (ml/kg/min)) conducted on a bicycle ergometer using a standardised ergometer ramp protocol. Work rate was progressively increased with 10, 15, 20, or 25 Watt per minute, depending on the estimated physical capacity of the participant prior to the test. Stopping criteria were physical exhaustion, <60 complete pedal rotations on the bike per min, or for safety reasons [31].

### 2.7 Cognitive and neuropsychiatric function

Additional to the cognitive screening (MMSE), an extensive standardised neuropsychological test battery was used to measure memory, attention & psychomotor speed, language, and executive function [32,33]. Memory included the Rey Auditory Verbal Learning Test (RAVLT) immediate recall, delayed recall, and recognition [34,35] and Visual Association Test (VAT) A (short version) [36]. Language was evaluated with the VAT naming and 1-minute animal fluency [37,38]. Attention and psychomotor speed included the Trail Making Test (TMT) A [39], Stroop Colour Word Test (SCWT, mean of 1 and 2) [[40], [41], [42]], Letter Digit Substitution Test (LDST) [43], and Digit span forward [44]. Executive functioning was assessed with TMT B/A index (TMTB/TMTA), SCWT interference score (card3/[card1 + card2]/2)), and Digit span backward. Cognitive test scores at baseline and follow-up were standardised into z-scores using healthy controls from the Heart-Brain Consortium as reference group [33], calculated as: Z-score = (test score – mean score reference group) / SD reference group [45]. Scores of the RAVLT recognition part, TMT, and SCWT, were inverted for higher scores to indicate better cognitive performance, calculated as: inverted z-score = Z*−1. The z-score of the cognitive domain was calculated as the average z-score of the tests in that domain. Subsequently, global cognitive function was calculated as the average z-score across all four domains. In addition, the Geriatric Depression Scale (GDS) [46] and the Starkstein Apathy Scale [47] were performed to test symptoms of depression and apathy, respectively, with higher scores reflecting more symptoms.

### 2.8 Plasma markers

Non-fasting EDTA plasma was obtained by venipuncture. The plasma was centrifugated at 1800 g for 10 min at room temperature and subsequently stored at −80 °C in aliquots of 0.5 mL in Sarsedt polypropylene tubes. Triglycerides, Hb1Ac, low-density lipoprotein (LDL) cholesterol, high-density lipoprotein (HDL) cholesterol, C-reactive protein (CRP), homocysteine, and thyroid-stimulating hormone (TSH), were assessed according to procedures also used in routine clinical chemistry. Brain-derived neurotrophic factor (BDNF) [48] and vascular endothelial growth factor (VEGF) [49] were assessed as described previously.

The samples for amyloid-beta (Aβ) 42, Aβ40, glial fibrillary acidic protein (GFAP), and neurofilament light protein (NfL) were briefly thawed at room temperature and centrifuged at 10,000 g for 10 min before usage to avoid interference of sample debris with the measurements. The Simoa™ Neurology 4-plex E Kit (Quanterix, Billerica, USA) on the Simoa HDX analyzer (Quanterix) was used to measure plasma levels of Aβ40, Aβ42, GFAP and NfL. Samples of each patient were analyzed in the same run, in duplicate with 1:4 automated on-board sample dilution, according to manufacturer’s instructions. Intra-assay coefficient of variation ( %CV) was 3.9 % for Aβ42, 3.0 % for Aβ40, 6.6 % for GFAP, and 5.0 % for NfL.

### 2.9 Statistical analysis

Original power calculations indicated that in order to detect an effect of the exercise program on CBF, a total sample size of 74 patients (37 patients per group) was needed for an effect size of 0.6 (effect size corresponding to a difference in mean change in CBF of 3 ± 5 mL/100 g/min) with a significance level of 0.05 and statistical power of 80 % [14]. Due to COVID-19 our participant inclusion was limited and the proposed sample size according to these calculations was not reached. Population characteristics were compared between the control and exercise group with chi-square or independent *t*-tests where appropriate. Missing data were not imputed.

To investigate the effect of aerobic exercise on CBF, intention-to-treat repeated measures analysis of variance (ANOVA) was used where the randomisation group (exercise or control) was entered as between-group variable and follow-up time as within-group variable, and CBF measurements as primary outcome. The secondary and exploratory outcome measures – VO2max, cognitive function, symptoms of depression and apathy, brain volumes, and plasma markers – were separately entered as dependent variables. Partial eta squared (η²) was used to represent effect sizes for our repeated measures ANOVA analyses, with values of 0.01 indicating small effects, 0.06 medium effects, and 0.14 large effects.

In addition, a per-protocol and several sensitivity analyses were performed for the outcomes CBF, cardiorespiratory fitness, and global cognition. In the per protocol analyses we excluded participants who completed <90 % (i.e. 38 out of 42) of the exercise sessions, and participants who did not adhere to the follow-up duration. The cut-off for prolonged follow-up duration was 18 weeks, describing the timeframe of 14 weeks intervention plus a maximum of 14 days previously to the first exercise session and following the final exercise session in which the baseline and follow-up measurements were to be performed.

In sensitivity analyses we evaluated the effect of population characteristics that differed significantly between the exercise and control group at baseline and the effect of load of small vessel disease features by including these as covariates into our models. Second, to assess the potential influence of outliers, z-scores were calculated for each variable, and participants with a z-score lower than −3SD or higher than +3SD were removed from analyses. Third, to assess the effect of maximal versus submaximal cardiopulmonary exercise test performance, participants were included only when they had reached their maximal exercise capacity during the baseline as well as the follow-up test. Maximal exercise capacity was defined as a heart rate ≥85 % of the estimated maximal heart rate (estimated as 220-age), and a respiratory exchange ratio (RER) reached ≥1.05 at peak exercise [50].

Regression analyses at baseline were performed to assess cross-sectional relationships between VO2max, grey matter CBF and cognition, adjusting for educational level, age, and sex. We also performed longitudinal regression analyses to investigate whether change in VO2 max from baseline to follow-up (predictor) was related to 1) change in grey matter CBF or 2) change in cognitive function and to investigate whether change in grey matter CBF (predictor) was associated with change in cognitive function, irrespective of group allocations.

Last, we expected that cardiac output or cardiorespiratory fitness (VO2max) at baseline affects the magnitude of the response to the exercise intervention. Therefore, interaction analyses were performed between randomisation group with either cardiac output, or VO2max by separately adding the interaction terms group*CO*time or group*VO2max*time to our models. In the presence of a significant interaction (*p* < 0.10), stratified analyses were performed with stratification at the median for high vs. low cardiac output and/or high vs. low VO2max (with a significance level of *p* < 0.05).

## 3 Results

### 3.1 Participants

**Fig. 1:**
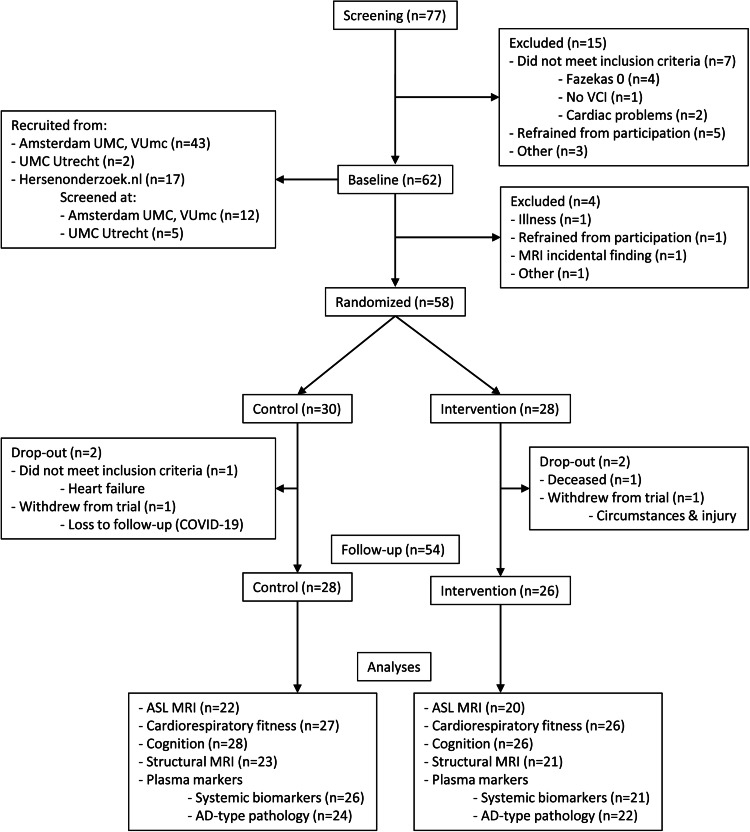
Flow diagram for the randomised controlled trial. Abbreviations: VCI = Vascular Cognitive Impairment, UMC = University Medical Centre, ASL MRI = arterial spin labelling magnetic resonance imaging, AD = Alzheimer’s Disease.

Seventy-seven subjects were screened to participate in the study. In total, 15 participants did not pass the screening, among which seven did not meet the inclusion criteria, five refrained from participation and three for other reasons (e.g. safety reasons during high-intensity exercise or COVID-19). Sixty-two participants underwent baseline measurements, of which 4 were excluded and 58 were randomised in either the control (*n* = 30) or the exercise (*n* = 28) group (Fig. 1). Sixteen (53 %) participants in the control group and 16 (57 %) participants in the exercise group were female. Participants’ mean±SD age at baseline was 67.2 ± 6.4 years in the control and 66.7 ± 7.2 years in the exercise group (Table 1). During the study two participants dropped out in each group. Fifty-four participants underwent follow-up measurements. Participants completed an average of 40 exercise sessions. Fig. 1 provides a flow chart of the study.

**Table 1:** Baseline characteristics of participants.

	**Total (*n*=58)**	**Control (*n*=30)**	**Exercise (*n*=28)**
Age, y (mean ± SD)	67.0 (6.7)	67.2 (6.4)	66.7 (7.2)
Women, n ( %)	32 (55.2)	16 (53.3)	16 (57.1)
Education, y (mean ± SD)	15.2 (4.5)	15.9 (4.6)	14.4 (4.3)
MMSE (median ± IQR)	29 (28–30)	29 (28–30)	29 (28–30)
Time baseline to follow-up, months (mean ± SD)	4.2 (0.7)	3.8 (0.5)	4.6 (0.6)
**Vascular risk factors**			
Hypertension*, n ( %)	39 (72.2)	17 (58.6)	22 (88.0)
Hypercholesterolemia*, n ( %)	39 (69.6)	17 (56.7)	22 (84.6)
Diabetes mellitus, n ( %)	14 (24.1)	9 (30.0)	5 (17.9)
Currently smoking, n ( %)	5 (8.6)	1 (3.3)	4 (14.3)
Alcohol ≥15 units, n ( %)	4 (6.9)	1 (3.3)	3 (10.7)
BMI ≥30 kg/m^2, n ( %)	15 (25.9)	6 (20.0)	9 (32.1)
**MRI measurements**			
**Brain MRI**	**Total (*n*=58)**	**Control (*n*=30)**	**Exercise (*n*=28)**
SVD ≥1*, n ( %)	25 (46.3)	12 (42.9)	13 (50.0)
Presence of microbleeds*, n( %)	17 (30.9)	11 (39.3)	6 (22.2)
Moderate to severe PVS, n ( %)	2 (3.4)	2 (6.7)	0 (0.0)
Fazekas ≥2*, n ( %)	35 (64.8)	20 (71.4)	15 (57.7)
WMH volume*^,^†, ml (mean ± SD)	0.74 (0.70)	0.79 (0.67)	0.66 (0.76)
Presence of lacunar infarcts*, n ( %)	8 (14.8)	5 (17.9)	3 (11.5)
Presence of cortical & subcortical infarcts*, n ( %)	9 (16.7)	4 (14.3)	5 (19.2)
**Cardiac MRI**	**Total (*n*=43)**	**Control (*n*=21)**	**Exercise (*n*=22)**
Cardiac Output, L/min (mean ± SD)	5.6 (1.0)	5.6 (1.0)	5.7 (1.1)

Abbreviations: SD = standard deviation, IQR = inter quartile range, MMSE = Mini Mental State examination, BMI = body mass index, SVD = cerebral small vessel disease, PVS = periventricular spaces, WMH = white-matter hyperintensities.

⁎ Due to missing data, hypertension: *n* = 54 (control =29, intervention=25), hypercholesterolemia: *n* = 56 (control=30, exercise=26), SVD: *n* = 54 (control=28, exercise=26), microbleeds: *n* = 55 (control =28, exercise=27), Fazekas: *n* = 54 (control=28, exercise=26), WMH volume: *n* = 56, (control=28, exercise=28), lacunar infarcts: *n* = 54 (control=28, exercise=26), cortical & subcortical infarcts: *n* = 54 (control =28, exercise=26).

† Log transformed and adjusted for intracranial volume.

### 3.2 ASL-MRI

**Fig. 2:**
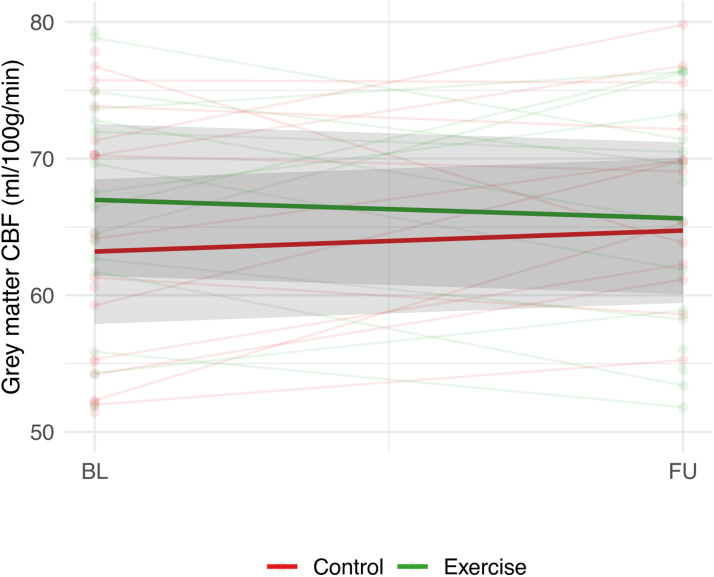
Change in CBF (ml/100 g/min) in the exercise and control group. Shaded grey areas provide confidence intervals. BL=Baseline, FU=Follow-up. CBF= Cerebral Blood Flow. P-value for group differences in change in CBF=0.38.

Change in grey matter CBF in ml/100 g/min from baseline to follow-up did not differ between the exercise (−1.4 (SE 2.4)) and the control group (1.5 (SE 2.3), *p* = 0.38) (Fig. 2), nor did spatial CoV (*p* = 0.38) (Table 2). Regional CBF changes in frontal, temporal, occipital or parietal grey matter also did not differ between the exercise and the control group (all *P* > 0.05) (Supplementary table A.1).

**Table 2:** Primary outcome.

	Control group		Exercise group			
Outcome	Baseline mean (SD)	Within-group change mean (SE)	Baseline mean (SD)	Within-group change mean (SE)	P-value	η²
ASL MRI*	*n* = 22		*n* = 20			
Grey matter CBF (ml/100 g/min)	63.2 (9.9)	1.5 (2.3)	67.0 (11.6)	−1.4 (2.4)	0.38	0.02
Spatial CoV† ( %)	1.71 (0.06)	0.03 (0.01)	1.71 (0.06)	0.01 (0.01)	0.38	0.02

Abbreviations: SD = standard deviation, SE = standard error, η² = partial eta squared, ASL MRI = arterial spin labelling magnetic resonance imaging, CBF = cerebral blood flow.

⁎ Exclusion of 9 participants due to suboptimal quality of ASL scan (1), vascular artefacts with little tissue perfusion signal (5) or unreliably high CBF values (3).

† Log transformed. P-value for group differences in change according to RM ANOVA.

### 3.3 Fitness

**Fig. 3:**
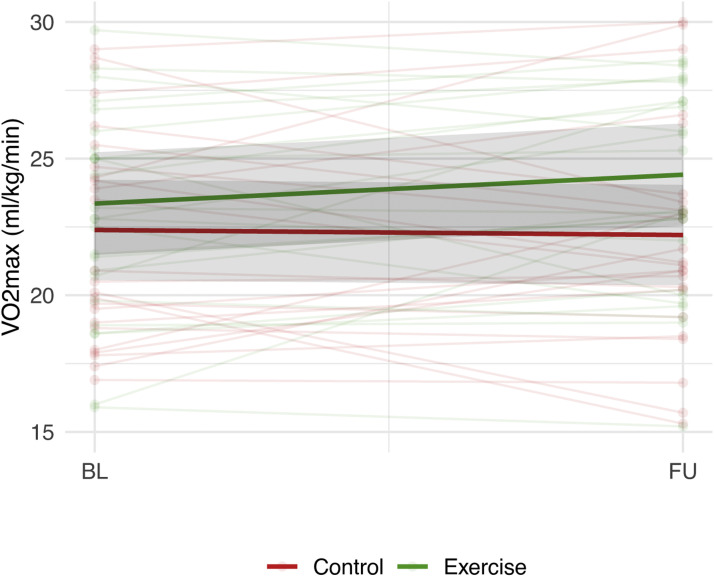
Change in VO2max (ml/kg/min) in the exercise and control group. Shaded grey areas provide confidence intervals. BL=Baseline, FU=Follow-up. CBF= Cerebral Blood Flow. P-value for group differences in change in VO2max=0.17.

The exercise group showed a non-significant increase in their VO2max (ml/kg/min) of 1.1 (SE 0.6) compared to −0.2 (SE 0.6) in the control group (*P* = 0.17; Fig. 3, Table 2).

### 3.4 Cognition

**Fig. 4:**
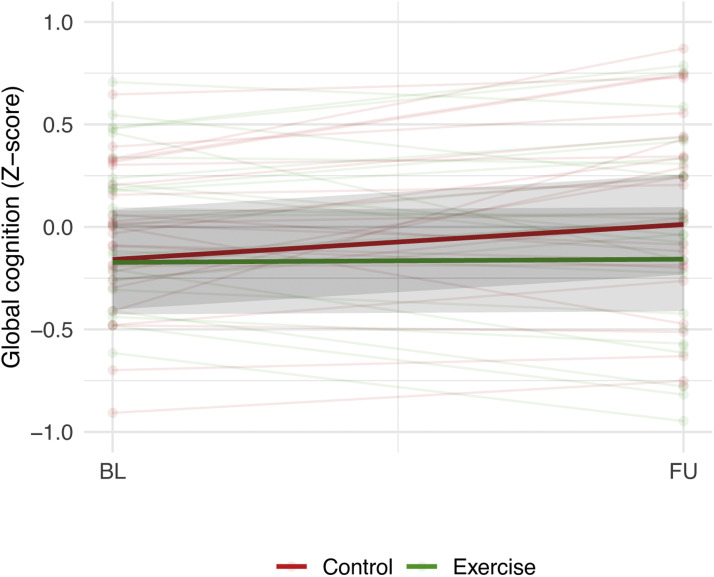
Change in global cognitive function in the control and exercise group. Shaded grey areas provide confidence intervals. BL=Baseline, FU=Follow-up. P-value for group differences in change in global cognition z-score=0.08.

Changes in z-scores for the cognitive domains (memory, language, attention and psychomotor speed, and executive function) and for symptoms of depression and apathy did not differ significantly between the exercise and the control group (all *P* > 0.05) (Table 3), nor did the change in z-score for global cognitive function (*p* = 0.08) (Fig. 4).

**Table 3:** Secondary outcomes.

	Control group		Exercise group			
Outcome	Baseline mean (SD)	Within-group change mean (SE)	Baseline mean (SD)	Within-group change mean (SE)	P-value	η²
Fitness (ml/kg/min)	*n* = 27		*n* = 26			
VO2max (ml/kg/min)	22.4 (5.1)	−0.2 (0.6)	23.4 (4.2)	1.1 (0.6)	0.17	0.04
**Cognitive function (Z-score)**	***n*=28**		***n*=26**			
Global cognitive function	−0.16 (0.50)	0.17 (0.06)	−0.17 (0.79)	0.02 (0.06)	0.08	0.06
Memory	0.01 (1.05)	0.08 (0.15)	−0.09 (1.22)	−0.06 (0.15)	0.51	0.01
Language	−0.17 (0.47)	0.06 (0.11)	−0.35 (0.82)	0.08 (0.12)	0.88	0.00
Attention and psychomotor speed	−0.08 (0.68)	0.10 (0.09)	−0.12 (0.90)	0.02 (0.10)	0.57	0.01
Executive function	−0.39 (0.82)	0.45 (0.17)	−0.14 (0.81)	0.02 (0.18)	0.09	0.06

Abbreviations: SD = standard deviation, SE = standard error, η² = partial eta squared.

P-value for group differences in change from baseline to follow-up in outcomes according to RM ANOVA (P-interaction time*randomisation group).

### 3.5 Structural MRI

Change in WMH volume (ml, log transformed) did not differ between the exercise (0.05 (SE 0.05)) and the control group (−0.02 (SE 0.05), *p* = 0.30), nor did white matter, grey matter or cerebrospinal fluid volumes (all *P* > 0.05) (Supplementary table A.2).

### 3.6 Plasma markers

Triglycerides in mmol/L increased in the exercise group by 0.35 (SE 0.17) in contrast with the control group (−0.12 (SE 0.15); *p* = 0.04). None of the other plasma markers (Hb1Ac, LDL-cholesterol, HDL-cholesterol, CRP, homocysteine, thyroid stimulating hormone, BDNF, VEGF, Aβ42, Aβ40, Aβ42/40, GFAP, and NfL) showed a significant difference in change between the exercise and the control group (all *P* > 0.05) (Supplementary table A.2).

### 3.7 Per protocol analysis

When we excluded three participants who did not complete 90 % (≥38) of the exercise sessions and four participants who did not adhere to the follow-up duration of 18 weeks (of which two due to holiday and two due to injury unrelated to the intervention), we found an increase in VO2max (ml/kg/min) in the exercise group of 1.8 (SE 0.7) compared to −0.2 (SE 0.6) in the control group (*p* = 0.04). Otherwise the analysis showed a similar pattern of results as intention-to-treat analyses (Supplementary table A.3).

### 3.8 Sensitivity analyses

The exercise and the control group significantly differed in prevalence of hypertension (control group=58.6 %, exercise group=88.0 %, *p* = 0.02) and hypercholesterolemia (control group=56.7 %, exercise group=84.6 %, *p* = 0.02), but did not differ in presence of SVD features. Analyses with these variables added as covariates, as well as analyses with removal of participants who did not reach their maximal exercise capacity, gave a similar pattern of results as the intention-to-treat analysis (data not shown). Removal of outliers showed a significant difference in change in global cognition between the exercise (−0.01 (SE 0.06)) and the control group (0.17 (SE 0.06), *p* = 0.04), and otherwise gave a similar pattern of results (data not shown).

Linear regression analyses of baseline data showed that a higher VO2max was related to a better global cognitive function (unstandardised β=0.3, *p* = 0.01) and to better executive function (unstandardised β=0.3, *p* = 0.007), while adjusting for age, sex and education (Supplementary table A.4). There were no cross-sectional associations between VO2max and grey matter CBF (*p* = 0.84, adjusted for age and sex), or between grey matter CBF and global cognitive function (*p* = 0.54, adjusted for age, sex, and education).

Regression analysis of changes over time irrespective of group allocations did not yield significant associations between VO2max and grey matter CBF or between VO2max and cognitive function, nor between grey matter CBF and cognitive function (data not shown).

There was no significant interaction present between time, randomisation group and cardiac output on CBF, cardiorespiratory fitness, or global cognition (all P-interaction group*CO*time >0.10), nor between time, randomisation group and VO2max on these outcomes (all P-interaction group*VO2max*time >0.10, data not shown).

## 4 Discussion

This randomised controlled trial with a 14-week aerobic exercise program in patients with VCI was unable to show improvements in our primary outcome global grey matter perfusion. The exercise program did not show any beneficial effects on cardiorespiratory fitness, cognitive function, symptoms of depression or apathy, nor had an effect on WMH, brain volumes, or blood biomarkers for metabolic disease, inflammation, growth factors, Alzheimer’s Disease, or neurodegeneration. Our per protocol analysis showed that the exercise group improved their cardiorespiratory fitness, underlining that adherence to the program is a crucial prerequisite for a physical activity intervention to have effect.

Previous trials investigating the effect of exercise on CBF have largely been performed in other populations and show mixed results. In line with the absence of effect found in our study, no effect of an exercise program on grey matter CBF was found in healthy men at 8 weeks follow-up [6] as measured with ASL-MRI. Other trials also reported that exercise interventions did not have significant effects on whole-brain CBF (grey and white matter) after 16 weeks in Alzheimer’s Disease patients [8] or after 12 weeks in healthy older adults [51], both as measured with ASL-MRI. In all three trials, participants performed 3 exercise sessions per week with session duration ranging from 50 to 60 min, and exercise intensity being defined differently (as 50–75 % of maximal heart rate [51], 70 % of maximal workload [6], or 70–80 % of heart rate reserve [8]. Two RCTs with a longer follow-up duration of 1 year and an identical progressive exercise program – starting with 3 exercise sessions per week building up to 5 sessions per week, sessions of 25–30 min with training intensity ranging from 75 to 95 % of maximal heart rate – both using transcranial Doppler ultrasound (TCD), showed that an exercise program increased whole-brain CBF in healthy older adults [52] and in patients with Mild Cognitive Impairment [7]. The usage of different measurement modalities could explain the discrepancy in results. Blood flow velocity measurements from TCD have been found not to be fully associated with cerebral perfusion measurements from ASL-MRI [53]. Another explanation could be the longer treatment duration, with the 14 weeks treatment duration in our trial being too short to find effects of exercise on grey matter CBF, compared to a year of treatment duration. We initially hypothesized that CBF would show rapid changes in response to exercise. However, we can speculate that exercise may only show positive effects on perfusion after longer periods of consistent training, through prevention of further vascular damage rather than showing direct improvements.

Notably, in all these trials, an increase in VO2max was found after the exercise intervention, which was not present in our intention-to-treat analysis. However, in our per protocol analysis, in which we excluded participants who did not complete 90 % of the exercise sessions and participants with extreme follow-up durations, there was a significant effect of the intervention on cardiorespiratory fitness. An increase in VO2max as small as 1.0ml/kg/min has been reported to lead to clinically important improvements in cardiac outcomes and overall survival [54]. In our per protocol analysis, the exercise group showed a mean increase in VO2max of 1.8ml/kg/min. Despite the increase in VO2max, an effect on grey matter CBF remained absent. The discussed trials with shorter follow-up durations of 8 up to 16 weeks [[6], [8], [51]] also did not find effects on grey matter CBF or whole-brain CBF, despite increases in VO2max.

In addition, our cross-sectional analysis also did not demonstrate any association between VO2max and grey matter CBF. We can speculate that aerobic exercise does not impact cerebral perfusion, but does influence cerebrovascular reactivity or oxygen extraction in the brain, which have been shown to be altered by ageing and neurological disease [55,56]. ASL MRI measures perfusion in the brain and does not allow for measurements of reactivity or oxygen extraction. Cerebrovascular reactivity might show small improvements in response to exercise that can be measured by other MRI sequences, such as fMRI [57]. As an alternative hypothesis the mechanisms underlying the relationship between physical activity and cognitive functioning may not be mediated through CBF. Instead, physical activity may exert its effects on cognitive function through other pathways, such as neurogenesis or improved alertness.

One randomised controlled trial has been performed with VCI patients in which the effect of exercise on cognition was investigated [9]. In contrast to our study, the investigators found small but significant improvements on the memory and language domains after 6 months of exercise, almost double our follow-up duration, as measured with the ADAS-Cog scale [9]. We included both VCI patients with Subjective Cognitive Decline, i.e. subjective complaints but no objectifiable cognitive impairment on neuropsychologic testing, and with Mild Cognitive Impairment. Therefore, the population in our study scored cognitively relatively good at baseline, scoring only slightly lower than the healthy controls from the Heart-Brain Consortium reference group. This could diminish the possibilities for improvement on cognitive performance in this patient group. We found a positive association at baseline between cardiorespiratory fitness (i.e. VO2max) and global cognition, providing further support of the well-described relationship between exercise and cognitive function [58]. This association was mostly attributable to the executive function domain, which has been found to be influenced by exercise in older adults in systematic reviews [59,60]. Our sensitivity analyses showed a counterintuitive improvement in cognitive function in the control group compared to the exercise group, which we expect to be a chance finding due to multiple testing.

We observed that 14 weeks of exercise had no impact on WMH changes. In a longitudinal study, small associations were found between decreased physical activity levels and progression of WMH after 3 years [61]. Other studies following natural progression of WMH demonstrate changes over time periods of 2–5 years in community-based [62] and population-based [63] studies in middle-aged and older adults, respectively. Thus we cannot expect WMH to have changed over our follow-up time, however, consistent exercise might delay WMH growth over a longer period of time [61]. Our follow-up time was also too short to detect changes on other volumetric measures [64].

Contrary to our findings, positive effects of exercise on metabolic markers have been consistently reported by reviews [11,65]. We found a counterintuitive significant increase in triglycerides in participants in the exercise group, compared to the control group, while this metabolic marker has been found to decrease with shorter term exercise interventions (<16 weeks). The greatest benefits of exercise on lipid profile in adults are found in high-intensity exercise programs [11]; exercise intensity in our population might have been insufficient to obtain measurable effects of the intervention on metabolic markers. In addition, triglycerides in plasma are obtained from fats through nutrition [11], which we did not monitor. Therefore, nutritional behaviour could partially explain the results. Finally, just as with global cognition, this result could be a chance finding due to multiple testing.

Among growth factors, BDNF and VEGF have long been described as mediating factors between exercise and brain function [12]. More recently it has been found in systematic reviews that BDNF increases or remains the same after exercise interventions in elderly people [66] and varying results have been found in patients with Mild Cognitive Impairment and Alzheimer’s Disease [67]. In an RCT in VCI patients, an exercise program resulted in an increase in BDNF levels in females, while BDNF decreased in males [68]. The differing results could partially be explained by sex differences in responses to exercise [68]. However, as with the metabolic markers, exercise intensity could have been insufficient to obtain measurable changes in growth factors.

Few randomised controlled trials have investigated the effect of exercise on plasma Aβ or on plasma NfL. One RCT including Mild Cognitive Impaired patients found a non-significant decrease of plasma Aβ42 after 6 months of exercise [69], while we found a non-significant increase in plasma Aβ42. A 16-week aerobic exercise program was not able to reduce serum NfL concentrations in patients with mild Alzheimer’s Disease [70], similarly to our intervention which did not decrease plasma NfL.

Limitations of this study include the small sample size with heterogeneous patients and therefore limited statistical power. COVID-19 severely impacted recruitment of our study and we did not reach the sample size needed according to our power calculations (74 participants in total). We originally powered the study to demonstrate an effect size of 0.60. We were only able to include 58 participants, of which 42 could be analysed for our primary outcome measure. This implies that, assuming a similar effect size to originally estimated, we had a power of 0.60 for the primary outcome, and 0.73 for the secondary outcomes. Moreover, our sample size is in the same order of magnitude or even larger compared to other RCTs that investigate the effect of aerobic exercise on CBF, with sample sizes ranging from 10 [69] to a maximum of 29 [7] participants in each group.

Several other limitations are acknowledged in this study. First, as mentioned previously, the intervention duration of 14-weeks may have been too short to measure changes in perfusion or improvements in cognitive function. Second, our exercise and control group showed some imbalances in cardiovascular risk factors despite our randomised design by strata of CDR, age, gender, and centre. Although sensitivity analyses were performed including those factors that showed significantly different (hypertension and hypercholesterolemia) it is possible that the higher number of other cardiovascular risk factors (higher frequency of smokers, alcohol intake, and BMI) present in the exercise group may have impacted our findings. Third, our population was cognitively relatively healthy at baseline, hampering generalization of our results to more severely affected VCI patients. Fourth, physical activity or exercise behaviour of the control group was not monitored. Although regular participation in an exercise program was an exclusion criterion for eligibility of this study and participants were instructed to maintain their habitual activities, we cannot exclude that exercise activities in the control group may have led to smaller group differences. Fifth, adherence to the interventions exercise frequency was measured by means of self-report limiting the infallibility of the exercise adherence measurements. Lastly, we used single post labelling delay when acquiring ASL images that can be influenced by transit time artefacts, whilst arterial transit time may be lengthened in some cerebrovascular disease patients [26]. A multi-delay protocol could improve accuracy of CBF measurements in this group.

Future studies should involve bigger sample sizes for sufficient power to demonstrate an effect of aerobic exercise on change in CBF. Bigger sample sizes could be obtained for example by using a multicentre design, and by the use of less stringent exclusion criteria (e.g. also including participants with more severe vascular cognitive impairment). Objective ways to monitor adherence to exercise intensity as well as frequency should be considered (e.g. by the use of a bicycle ergometer) to be able to investigate a dose response relationship between exercise, physiological brain variables, and cognition. Additionally, information about reasons for non-adherence, as well as whether supervision levels impacted results, is important for assessing the feasibility of exercise programs. Lastly, more sensitive measures for CBF could be used to detect subtle changes that may not have been captured by the current study’s methods, including measurements that allow for analysis of cerebrovascular reactivity as well as oxygen extraction fraction to provide valuable mechanistic insight.

A major strength of this study is the randomised controlled design including VCI patients, a group which is underrepresented in research, and the acquisition of longitudinal ASL-MRI measurements. To date, this is the only trial investigating CBF responses to an exercise program in this patient group. Another strength is the low dropout rate, with only 2 dropouts in both groups. Also, we broadly explored the effect of the exercise program on various health outcomes, among which an extensive cognitive testing battery and plasma Aβ markers. Last, the extensiveness of our per protocol and sensitivity analyses addressing many possible confounding factors aids in understanding the biological effects of exercise on the CBF parameters.

To conclude, in this randomised controlled trial a 14-week exercise program showed no increase in CBF or improvement in cognition in patients with VCI. In those participants who adhered to the protocol guidelines, the aerobic exercise program significantly improved cardiorespiratory fitness. Future research should include larger sample sizes, longer intervention durations, and include alternative measurement techniques for CBF and/or vascular reactivity.

## Funding sources

This work is part of the Heart-Brain Connection crossroads (HBCx) consortium of the Dutch CardioVascular Alliance (DCVA). HBCx has received funding from the Dutch Heart Foundation under grant agreements 2018–28 and CVON 2012–06. Hersenonderzoek.nl is funded by ZonMw-Memorabel (project no.73305095003), a project in the context of the Dutch Deltaplan Dementie, Gieskes-Strijbis Foundation, the Alzheimer’s Society in The Netherlands, and Brain Foundation Netherlands.

## Disclosures

Research programs of Wiesje van der Flier have been funded by ZonMW, NWO, EU-JPND, EU-IHI, Alzheimer Nederland, Hersenstichting CardioVascular Onderzoek Nederland, Health∼Holland, Topsector Life Sciences & Health, stichting Dioraphte, Gieskes-Strijbis fonds, stichting Equilibrio, Edwin Bouw fonds, Pasman stichting, stichting Alzheimer & Neuropsychiatrie Foundation, Philips, Biogen MA Inc, Novartis-NL, Life-MI, AVID, Roche BV, Fujifilm, Eisai, Combinostics. WF holds the Pasman chair. WF is recipient of ABOARD, which is a public-private partnership receiving funding from ZonMW (#73,305,095,007) and Health∼Holland, Topsector Life Sciences & Health (PPP-allowance; #LSHM20106). WF, GJB and EEB are recipients of TAP-dementia (www.tap-dementia.nl), receiving funding from ZonMw (#10,510,032,120,003) in the context of Onderzoeksprogramma Dementie, part of the Dutch National Dementia Strategy. TAP-dementia receives co-financing from Avid Radiopharmaceuticals and Amprion. Gieskes-Strijbis fonds also contributes to TAP-dementia. WF has been an invited speaker at Biogen MA Inc, Danone, Eisai, WebMD Neurology (Medscape), NovoNordisk, Springer Healthcare, European Brain Council. WF is consultant to Oxford Health Policy Forum CIC, Roche, Biogen MA Inc, and Eisai. WF is member of steering committee of NovoNordisk evoke/evoke+. WF participated in advisory boards of Biogen MA Inc, Roche, and Eli Lilly. All funding is paid to her institution. WF is member of the steering committee of PAVE, and Think Brain Health. WF was associate editor of Alzheimer, Research & Therapy in 2020/2021. WF is associate editor at Brain. MJPvO is a non-paid member of the steering committee of the APPRICORN trial of Alnylam and his research group receives research support from Philips. MD and HM are founded by the Dutch Heart Foundation [03–004–2020-T049]. HM is supported by the Eurostars-2 joint programme with co-funding from the European Union Horizon 2020 research and innovation programme (ASPIRE E!113,701), provided by The Netherlands Enterprise Agency (RvO), and by the EU Joint Program for Neurodegenerative Disease Research, provided by The Netherlands Organisation for health Research and Development and Alzheimer Nederland (DEBBIE JPND2020–568–106).

## CRediT authorship contribution statement

**Liz R. van Hout:** Writing – review & editing, Writing – original draft, Formal analysis. **Justine Moonen:** Writing – review & editing, Writing – original draft, Supervision. **Annebet E. Leeuwis:** Writing – review & editing, Investigation. **Juliette van Alphen:** Writing – review & editing, Investigation. **Mathijs Dijsselhof:** Writing – review & editing, Visualization, Software. **Raquel P. Amier:** Writing – review & editing, Investigation. **Frederik Barkhof:** Writing – review & editing, Visualization, Software. **Esther E. Bron:** Writing – review & editing, Visualization, Software. **Doeschka A. Ferro:** Writing – review & editing, Investigation. **Alexander G.J. Harms:** Writing – review & editing, Visualization, Software. **Rosalie J. Huijsmans:** Writing – review & editing, Investigation. **Joost P.A. Kuijer:** Writing – review & editing, Visualization, Software. **Sanne Kuipers:** Writing – review & editing, Investigation. **Matthias J.P. van Osch:** Writing – review & editing, Visualization, Software. **Niels D. Prins:** Writing – review & editing, Investigation. **Marc B. Rietberg:** Writing – review & editing, Investigation. **Charlotte E. Teunissen:** Writing – review & editing, Investigation. **Henk Jan Mutsaerts:** Writing – review & editing, Visualization, Software. **Geert Jan Biessels:** Writing – review & editing, Supervision, Methodology, Funding acquisition, Conceptualization. **Wiesje M. van der Flier:** Writing – review & editing, Supervision, Methodology, Funding acquisition, Conceptualization.

## Declaration of competing interest

The authors declare that they have no financial or personal relationships with any organizations that could inappropriately influence the content of this article.

## Appendix Supplementary materials

Image, application 1

Image, application 2
