# Supplementary material for: The effect of aerobic exercise on cerebral perfusion in patients with vascular cognitive impairment, the Excersion-VCI randomised controlled clinical trial
Source: Cereb Circ Cogn Behav. 2025 May 24;8:100386. doi: 10.1016/j.cccb.2025.100386 (PMC12166751; doi:10.1016/j.cccb.2025.100386)
Supplement: Supplementary file 1 [file mmc1.docx]

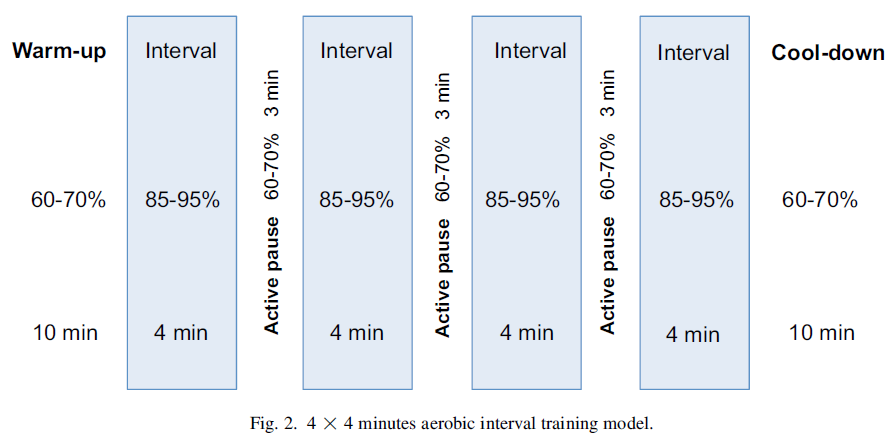


Supplementary figure A.1. 4x4 aerobic interval model. Percentages represent the workout intensity as % HRpeak. *Note*. Reprinted from “Design of the ExCersion-VCI study: The effect of aerobic exercise on cerebral perfusion in patients with vascular cognitive impairment.” Leeuwis et al. (2017). *Alzheimer’s & Dementia, 3*(2), p. 161.
