# Supplementary material for: The effect of aerobic exercise on cerebral perfusion in patients with vascular cognitive impairment, the Excersion-VCI randomised controlled clinical trial
Source: Cereb Circ Cogn Behav. 2025 May 24;8:100386. doi: 10.1016/j.cccb.2025.100386 (PMC12166751; doi:10.1016/j.cccb.2025.100386)
Supplement: Supplementary file 2 [file mmc2.docx]

# Appendix A

| **Supplementary table A.1: CBF in brain lobes** | | | | | | | |  |
| --- | --- | --- | --- | --- | --- | --- | --- | --- |
|  | **Control (n=22)** | | **Exercise (n=20)** | |  |  | |  |
| **Outcome (mean ml/100g/min)** | **Baseline**  **mean (SD)** | **Within-group change**  **mean (SE)** | **Baseline**  **mean (SD)** | **Within-group change**  **mean (SE)** | **P-value** | | **η²** |  |
| Occipital L | 63.7 (10.5) | 2.9 (2.4) | 70.1 (13.9) | -2.8 (2.6) | 0.12 | | 0.06 |  |
| Occipital R | 63.3 (10.2) | 2.2 (2.3) | 70.3 (12.8) | -2.4 (2.4) | 0.17 | | 0.05 |  |
| Temporal L | 60.0 (7.7) | 1.7 (2.2) | 61.9 (12.6) | -0.3 (2.3) | 0.54 | | 0.01 |  |
| Temporal R | 58.5 (9.4) | 1.3 (2.4) | 61.3 (11.6) | -1.5 (2.5) | 0.43 | | 0.02 |  |
| Parietal L | 63.8 (10.2) | 2.3 (2.2) | 69.9 (12.7) | -1.0 (2.3) | 0.31 | | 0.03 |  |
| Parietal R | 61.8 (9.5) | 1.2 (2.2) | 67.7 (11.4) | 0.2 (2.3) | 0.75 | | 0.00 |  |
| Frontal L | 65.9 (10.6) | 2.1 (2.6) | 70.8 (13.2) | -1.0 (2.8) | 0.43 | | 0.02 |  |
| Frontal R | 63.1 (10.0) | 1.5 (2.5) | 70.0 (12.3) | -0.8 (2.6) | 0.53 | | 0.01 |  |

Abbreviations: SD = standard deviation, SE = standard error, η² = partial eta squared, CBF = cerebral blood flow, L = left, R = right.

P-value for group differences in change from baseline to follow-up in outcomes according to RM ANOVA (i.e. P-interaction time*randomisation group).

| **Supplementary table A.2: Secondary outcome measures** | | | | | | | |
| --- | --- | --- | --- | --- | --- | --- | --- |
|  | **Control** | | **Exercise** | |  |  | |
| **Outcome** | **Baseline**  **mean (SD)** | **Within-group change**  **mean (SE)** | **Baseline**  **mean (SD)** | **Within-group change**  **mean (SE)** | **p-value** | | **η²** |
| **Symptoms of depression and apathy** | **n=28** |  | **n=26** |  |  | |  |
| Geriatric depression scale | 2.36 (1.89) | -0.39 (0.34) | 2.00 (1.88) | -0.19 (0.35) | 0.68 | | 0.00 |
| Starkstein apathy scale | 10.75 (3.50) | -0.18 (0.69) | 9.85 (5.45) | 0.12 (0.72) | 0.77 | | 0.00 |
| **Structural MRI (ml)*** | **n=23** |  | **n=21** |  |  | |  |
| GM volume | 630.1 (58.5) | 12.0 (10.2) | 618.7 (64.8) | 24.3 (10.7) | 0.41 | | 0.02 |
| WM volume | 432.6 (44.6) | 13.5 (10.5) | 434.3 (57.4) | 1.0 (11.0) | 0.42 | | 0.02 |
| WMH volume** | 0.71 (0.61) | -0.02 (0.05) | 0.69 (0.84) | 0.05 (0.05) | 0.30 | | 0.03 |
| CSF volume | 314.2 (45.7) | -24.7 (3.7) | 326.5 (68.4) | -23.4 (3.9) | 0.81 | | 0.00 |
| **Systemic plasma markers** | **n=26** |  | **n=21** |  |  | |  |
| Triglycerides | 1.82 (0.85) | -0.12 (0.15) | 1.63 (0.56) | 0.35 (0.17) | 0.04* | | 0.09 |
| HbA1c** | 1.63 (0.10) | -0.001 (0.01) | 1.60 (0.05) | 0.02 (0.01) | 0.12 | | 0.05 |
| Total cholesterol | 4.96 (1.05) | -0.09 (0.10) | 4.73 (1.35) | 0.06 (0.11) | 0.32 | | 0.02 |
| LDL-cholesterol † | 2.58 (0.93) | -0.03 (0.09) | 2.32 (1.16) | -0.03 (0.11) | 0.99 | | 0.00 |
| HDL-cholesterol | 1.56 (0.61) | -0.01 (0.04) | 1.54 (0.41) | 0.01 (0.05) | 0.71 | | 0.00 |
| CRP | 3.30 (2.32) | -0.35 (0.24) | 2.46 (0.91) | -0.28 (0.26) | 0.84 | | 0.00 |
| Homocysteine † | 10.79 (3.52) | 0.09 (0.45) | 12.40 (3.41) | -0.09 (0.49) | 0.79 | | 0.00 |
| TSH | 1.94 (0.89) | -0.11 (0.11) | 2.02 (1.22) | 0.08 (0.13) | 0.28 | | 0.03 |
| BDNF † | 1.92 (0.90) | 0.59 (0.45) | 2.72 (2.12) | 0.65 (0.47) | 0.94 | | 0.00 |
| VEGF** † | 1.57 (0.24) | 0.08 (0.04) | 1.53 (0.29) | 0.12 (0.04) | 0.55 | | 0.01 |
| **AD plasma markers** | **n=24** |  | **n=22** |  |  | |  |
| Aβ40 | 104.0 (29.47) | -2.40 (3.17) | 119.18 (48.31) | 1.19 (3.31) | 0.44 | | 0.01 |
| Aβ42 | 7.02 (2.13) | -0.33 (0.31) | 7.40 (2.92) | 0.26 (0.33) | 0.20 | | 0.04 |
| Aβ42/ Aβ40 | 0.07 (0.05) | -0.002 (0.002) | 0.06 (0.02) | 0.003 (0.002) | 0.10 | | 0.06 |
| GFAP** | 2.01 (0.26) | 0.003 (0.02) | 2.00 (0.20) | -0.02 (0.02) | 0.56 | | 0.01 |
| NfL** | 1.22 (0.21) | 0.003 (0.03) | 1.26 (0.27) | -0.03 (0.03) | 0.49 | | 0.01 |

Abbreviations: SD = standard deviation, SE = standard error, η² = partial eta squared, GM = grey matter, WM = white matter, WMH = white matter hyperintensities, CSF = cerebrospinal fluid, LDL-cholesterol = low-density lipoprotein cholesterol, HDL-cholesterol = high-density cholesterol, CRP = C-reactive protein, TSH = thyroid stimulating hormone, BDNF = brain-derived neurotrophic factor, VEGF = vascular endothelial growth factor, Aβ = Amyloid-beta, GFAP = glial fibrillary acidic protein, NfL = neurofilament light chain.

*All structural MRI markers are adjusted for intracranial volume.

**Log transformed.

†Due to missing data, LDL cholesterol: control=26, exercise=20; homocysteine: control=25, exercise=21; BDNF: control=24, exercise=22, VEGF: control=24, exercise=22.

P-value for group differences in change from baseline to follow-up in outcomes according to RM ANOVA (i.e. the P-interaction time*randomisation group).

| **Supplementary table A.3: Per protocol analyses – Adherence** | | | | | | |
| --- | --- | --- | --- | --- | --- | --- |
|  | **Control group** | | **Exercise group** | |  | |
| **Outcome** | **Baseline**  **mean (SD)** | **Within-group change**  **mean (SE)** | **Baseline**  **mean (SD)** | **Within-group change**  **mean (SE)** | **P-value** | **η²** |
| **ASL MRI** | **n=22** |  | **n=15** |  |  | |
| GM CBF (ml/100g/min) | 63.2 (9.9) | 1.5 (2.3) | 65.2 (12.4) | -1.0 (2.8) | 0.49 | 0.01 |
| **Fitness (ml/kg/min)** | **n=27** |  | **n=19** |  |  | |
| VO2max (ml/kg/min) | 22.4 (5.1) | -0.2 (0.6) | 23.0 (4.4) | 1.8 (0.7) | 0.04* | 0.09 |
| **Cognition** | **n=28** |  | **n=19** |  |  | |
| Global cognition | -0.16 (0.50) | 0.17 (0.06) | -0.21 (0.84) | -0.01 (0.07) | 0.05 | 0.08 |

Abbreviations and calculations as in Table 2 and 3.

P-value for group differences in change from baseline to follow-up in outcomes according to RM ANOVA (P-interaction time*randomisation group).

| **Supplementary table A.4: Cross-sectional association of VO2max and cognitive function at baseline** | | | |
| --- | --- | --- | --- |
| **Outcome variable** | **Unstandardised B (SE)** | **Beta** | **p-value** |
| Global cognition | 0.04 (0.02) | 0.3 | 0.014* |
| Memory | 0.04 (0.03) | 0.1 | 0.24 |
| Language | 0.03 (0.02) | 0.2 | 0.08 |
| Attention & psychomotor speed | 0.03 (0.02) | 0.2 | 0.20 |
| Executive function | 0.06 (0.02) | 0.3 | 0.007* |

Adjusted for education level, age, and sex.
